# Infant feeding practices at routine PMTCT sites, South Africa: results of a prospective observational study amongst HIV exposed and unexposed infants - birth to 9 months

**DOI:** 10.1186/1746-4358-7-4

**Published:** 2012-04-03

**Authors:** Ameena E Goga, Tanya Doherty, Debra J Jackson, David Sanders, Mark Colvin, Mickey Chopra, Louise Kuhn

**Affiliations:** 1 Medical Research Council, Tygerberg& Pretoria, Pretoria Regional Office, 1 Soutpansberg Road, Pretoria, Private Bag x385, Pretoria 0001, South Africa; 2 Department of Paediatrics and Child Health, Kalafong Hospital, Pretoria, South Africa; 3 Maromi Health Research, Inthuthuko Building (2nd Floor, HSRC), 750 Francois Rd, Durban, 4001, Private Bag X07, Dalbridge 4014, South Africa; 4 School of Public Health, University of the Western Cape, PB X17, Modderdam Road, Bellville, Cape Town 7535, South Africa; 5 UNICEF New York, UNICEF House, 3 United Nations Plaza, New York, NY 10017, USA; 6 Gertrude H. Sergievsky Center, Columbia University, 630 W 168 Street, New York, NY 10032, USA

**Keywords:** PMTCT, HIV and Infant Feeding, Breastfeeding, HIV-free survival, Formula Feeding

## Abstract

**Background:**

We sought to investigate infant feeding practices amongst HIV-positive and -negative mothers (0-9 months postpartum) and describe the association between infant feeding practices and HIV-free survival.

**Methods:**

Infant feeding data from a prospective observational cohort study conducted at three (of 18) purposively-selected routine South African PMTCT sites, 2002-2003, were analysed. Infant feeding data (previous 4 days) were gathered during home visits at 3, 5, 7, 9, 12, 16, 20, 24, 28, 32 and 36 weeks postpartum. Four feeding groups were of interest, namely exclusive breastfeeding, mixed breastfeeding, exclusive formula feeding and mixed formula feeding. Cox proportional hazards models were fitted to investigate associations between feeding practices (0-12 weeks) and infant HIV-free survival.

**Results:**

Six hundred and sixty five HIV-positive and 218 HIV-negative women were recruited antenatally and followed-up until 36 weeks postpartum. Amongst mothers who breastfed between 3 weeks and 6 months postpartum, significantly more HIV-positive mothers practiced exclusive breastfeeding compared with HIV-negative: at 3 weeks 130 (42%) versus 33 (17%) (p < 0.01); this dropped to 17 (11%) versus 1 (0.7%) by four months postpartum. Amongst mothers practicing mixed breastfeeding between 3 weeks and 6 months postpartum, significantly more HIV-negative mothers used commercially available breast milk substitutes (p < 0.02) and use of these peaked between 9 and 12 weeks. The probability of postnatal HIV or death was lowest amongst infants living in the best resourced site who avoided breastfeeding, and highest amongst infants living in the rural site who stopped breastfeeding early (mean and standard deviations: 10.7% ± 3% versus 46% ± 11%).

**Conclusions:**

Although feeding practices were poor amongst HIV-positive and -negative mothers, HIV-positive mothers undertake safer infant feeding practices, possibly due to counseling provided through the routine PMTCT programme. The data on differences in infant outcome by feeding practice and site validate the WHO 2009 recommendations that site differences should guide feeding practices amongst HIV-positive mothers. Strong interventions are needed to promote exclusive breastfeeding (to 6 months) with continued breastfeeding thereafter amongst HIV-negative motherswho are still the majority of mothers even in high HIV prevalence setting like South Africa.

## Background

The vicious cycle of malnutrition, infection and mortality necessitates close attention to nutrition to meet the fourth millennium development goal (MDG4). Pattern of feeding (as defined by the World Health Organization - see Table 1) is a significant predictor of child morbidity and mortality [1-4]. Compared with exclusive breastfeeding (EBF), predominant (PredBF), partial (ParBF) or not breastfeeding (NBF) are associated with a higher mortality risk in general [RR and 95% CI: 1.48 (1.13, 1.92); 2.85 (1.59, 5.10) and 14.40 (6.09, 34.05), respectively at 0 to 5 months and 3.86 (1.49, 9.29) for NBF at 6 to 23 months)], from diarrhea [RR 2.28 (0.85, 6.11), 4.62 (1.81, 11.77), 10.53 (2.80, 39.64) respectively at 0 to 5 months and 2.83 (0.15, 54.82) for NBF 6 to 23 months)] and pneumonia [RR 1.75 (0.48, 6.43); 2.49 (1.03, 6.04); 15.13 (0.61, 373.84) respectively for 0 to 5 months and 1.52 (0.09, 27.06) for NBF 6 to 23 months] [1]. Despite such benefits, breast milk (BF) can transmit HIV. Mixed breastfeeding carries the highest risk of transmission and EBF the lowest [5-7]. On a population level however, universal coverage with EBF for six months, and continued breastfeeding up to one year may prevent 13% of under-five deaths globally, even in the context of HIV [8].

**Table 1:** WHO feeding definitions

Exclusive breastfeeding (EBF)	Giving the infant breast milk only and any minerals, vitamins and prescribed medicines if needed, for the first six months
**Mixed breastfeeding (MBF)**	Giving the infant breast milk and other fluids And solids. MBF may be further classified into predominant breastfeeding and partial breastfeeding: **Predominant breastfeeding **(PredBF) means giving the infant breast milk and non-nutritive liquids **Partial breastfeeding (ParBF) **means feeding breast milk andnon-nutritive and nutritive liquids and solids

**Exclusive formula feeding (EFF)**	Giving the infant only commercial infant formula milk for the first six months of life

**Replacement feeding (RF)**	Refers to the process of feeding a child who is not receiving any breast milk a diet that provides all the nutrients the child needs until the child is fully fed on family foods. During the first six months a suitable breast milk substitute should be used and subsequently complementary foods made from appropriately prepared and nutrient-enriched family foods should be added

Thus, although breastfeeding is a significant child survival strategy, HIV-positive mothers cannot simply all be told to breastfeed [9,10]. The benefits of breastfeeding with antiretroviral (ARV) prophylaxis should be weighed against the risks of HIV transmission through breastfeeding [11]. The World Health Organization (WHO) currently recommends that national or sub-national authorities should decide whether health services will principally counsel and support HIV-infected mothers to breastfeed and receive ARV interventions, or avoid all breastfeeding, to improve HIV-free survival [12]. The WHO further advises that HIV-infected mothers should only give commercial infant formula milk as a breast milk substitute when specific conditions are met. Simple, consistent approaches and tools for infant feeding in the context of HIV have been developed [9], but routine challenges exist including poor quality or lack of counseling, or poor support for continued infant feeding, and poor application of tools to identify women who could avoid all breastfeeding [13-15]. Data from research settings in Mexico and Bangladesh show that infant feeding practices improve after implementing specifically designed home-based interventions [5,16,17]. However, data on feeding practices in the context of routine PMTCT programmes are sparse: Most published studies with a longitudinal design [18,19] document poor follow-up, whilst those with a case-control or cross-sectional or retrospective design [20-22] have an inherent selection or information bias. The former cannot produce generalizable results, whereas the latter results in misclassification of feeding practices, especially EBF. For example, a recent hospital-based case-control study from Uganda [21] reported significantly lower EBF in PMTCT mothers (65%) versus non-PMTCT mothers (98%), p < 0.001. However in this study, participants were aged 3-12 months, and it is not known whether cases and controls were of comparable ages as age affects feeding practice. Analysis of breastfeeding patterns also included HIV-positive women who chose to formula feed.

South Africa has particularly poor infant feeding practices in the general population. Recently, a multi-country community-based cluster randomised trial on breastfeeding promotion found that EBF prevalence (amongst mothers intending to breastfeed) at 12 weeks in intervention and control clusters in Burkina Faso, Uganda and South Africa, respectively were 77% versus 23% in intervention versus control cluster, Burkina Faso (Prevalence ratio 3.27, 95% CI 2.13, 5.03); 77% vs 34% in intervention vs control clusters, Uganda (Prevalence ratio 2.30, 95% CI 2.00, 2.65); and 8% vs 4% in intervention vs control clusters, South Africa (Prevalence ratio 1.98, 95% CI 1.30, 3.02) [23].

We describe feeding practices amongst HIV-positive and -negative women at three routine South African PMTCT sites at the start of the national PMTCT programme and investigate the association between 0-12 week feeding practice and HIV-free survival.

## Methods

### Study setting and context

Data are from a prospective observational cohort study (September 2002-August 2003) conducted to determine the operational effectiveness of the Routine National South African PMTCT programme. Three sites were purposively selected [24]: Paarl, a well-resourced commercial farming area with 11% antenatal HIV prevalence, infant mortality rate (IMR) at 40 per 1000 live births; Rietvlei, a poverty-stricken deep rural area with 26% antenatal HIV prevalence, IMR 99/1000 and Umlazi, a peri-urban area with 36% antenatal HIV prevalence, IMR 60/1000 [25,26].

The 2002-2003 routine PMTCT programme included single-dose nevirapine, delayed rupture of membranes and infant feeding counselling. In Paarl, five lay counsellors received eight hours of in-service training on HIV and infant feeding; in Umlazi and Rietvlei nurses and lay counsellors were trained in a standardised five-day PMTCT and infant feeding training course[13]. In Umlazi one of the three lay counsellorsspecialised in infant feeding. For HIV-positive women post-test counselling and post-natal PMTCT visits included infant feeding counselling. Non-breastfeeding HIV-positive women received free commercial infant formula from routine health services for six months. HIV-negative women received infant feeding counselling during post-test counselling sessions, and were scheduled to receive infant feeding support postpartum from routine child health services. Quality of PMTCT and infant feeding counselling was poorest in Paarl, and best in Umlazi [13]. Highly active antiretroviral treatment was not national policy in 2002-3.

### Study design and sampling

Maternal HIV status was determined by the routine PMTCT programme [24]. Consecutive HIV- positive and -negative pregnant women were enrolled (3:1) and followed up until 36 weeks postpartum.

### Study procedures and data collection

Trained data collectors conducted home interviews at recruitment, 3, 5, 7, 9, 12, 16, 20, 24, 28, 32 and 36 weeks. The recruitment interview gathered data on socio-demographics, perceived quality of antenatal feeding counselling, feeding intention and knowledge of PMTCT. From 3 to 36 weeks, data collectors asked whether the infant received any of 15 food and liquid items 'yesterday' (from sunrise yesterday until sunrise today) and for 3 days prior to 'yesterday'. During the 3 week interview, data on feeding during the first postnatal week were gathered. At 3, 24 and 36 weeks check questions asked whether infants had 'ever breastfed'. Data collectors did not counsel about feeding or PMTCT. No diaries were used to remind mothers about infant feeding practices.

### Data definitions

Table 2 explains how we operationalized the World Health Organization feeding definitions. Socioeconomic score (SES) was estimated using principal component factor analysis using six household assets (refrigerator, radio, television, stove, telephone/cell phone, car) and questions about food security. A weighted average was produced - items with greater variability (e.g. television) contributed to more score than items with lesser variability (e.g. radio). High socioeconomic score denotes people with more assets and food security. Counseling score was a composite measure created from reported antenatal infant feeding counseling. For HIV-positive women: was feeding ever discussed antenatally? (+4 if yes, -4 if no and 0 if don't know), number of times discussed (0 - none, 1 - once only, 2 - twice, 3-3 times and 4 if > 3 times) and whether the following topics were mentioned: risks of MTCT and breastfeeding (+4 if yes), different formula feeding and breastfeeding options (+4 if yes), risk of giving formula feeds (+4 if yes), how to make best feeding choice (+4 if yes), if the mother intended to breastfeed, then avoiding mixed feeding and stopping breastfeeding early (+4 for each), how women were helped to make a choice - if women were helped to make an appropriate choice (score = +12); if health staff recommended a suitable option (score = +8); if little/no help or guidance provided with choice (score = +4). If health staff simply told women to breastfeed, score = -4. Thus maximum score was +44 and minimum was -8. For HIV-negative women the scores were as follows: if the counsellor reportedly discussed the risks of giving formula feeds (+4), advised against mixed feeding (+4), discussed the risks of MTCT (-4), discussed different formula feeding options (-4), advised the mother to stop breastfeeding by 6 months (-4) and discussed feeding options, helping the mother to make a choice (-4). Thus the maximum score was +8 and minimum was -16.

**Table 2:** Operationalising the WHO feeding definitions during data analysis

Any breast milk	Infant given some breast milk during any or all or the 4 days prior to the home visit
**Any formula milk**	Infant given any formula milk during any or all of the 4 days prior to the home visit

**Other milk**	Breast milk if formula was the main milk given and vice versa

**Non-nutritive fluids**	Glucose-water, sugar-water, tea/juice, traditional medicines and over-the-counter medicine

**Breast milk**	**Exclusive breastfeeding (EBF)**- infant given **only **breast milk with or without prescribed medicines during all of the 4 days prior to the home visit. No other liquids or solids reportedly given during any of these 4 days.
	
	**Mixed breastfeeding (MBF) **- Infant given breast milk and other fluids (non-nutritive or nutritive, including formula milk) or solids during any or all of the 4 days prior to the home visit. This includes infants who received mainly formula milk but who lapsed into exclusive breastfeeding during any of the four days prior to the home visit. Can be divided into 2 groups, viz: • **Pred BF **- infant given breast milk and other non-nutritive liquids only, during one or all of the 4 days prior to the home visit. No formula milk or solids given. • **ParBF - **- infant given breast milk and solids and liquids (nutritive or non- nutritive) during any or all of the 4 days prior to the home visit.

**No breast milk**	**Exclusive formula feeding (EFF) **infant reportedly given **only **formula milk during all of the 4 days prior to the home visit. Breast milk and other solids or liquids reportedly not given during these 4 days.
	
	**Formula feeding with liquids and solids** **(MFF) **- infant reportedly given formula milk with other liquids and solids during any or all of the 4 days prior to the home visit. Breast milk not given during any of these 4 days.

**Longitudinal feeding variable with 4 mutually exclusive groups:** These were generated based on the predominant feeding pattern per woman during the 20 days for which feeding data were available from 3-12 weeks (i.e. 4 days prior to the 3, 5, 7, 9 and 12 weeks visits), and from 'ever breastfeeding' questions.	**Longitudinal exclusive breastfeeding** **[EBF(l)] **- the infant started out with exclusive breastfeeding and was still exclusively breastfeeding at 12 weeks. Exclusive breastfeeding defined as infant given **only** breast milk with or without prescribed medicines. Infants were excluded from this category if > 1 day of non-exclusive breastfeeding occurred between 0 and 12 weeks.
	
	**Longitudinal mixed breastfeeding [MBF(l)]**- infant received breast milk and other nutritive or non-nutritive substances for 2 days or more, at any time between 0 and 12 weeks.
	
	**Avoiding breastfeeding **- between 0 and 12 weeks infant only received breast milk for one day or did not receive breast milk at all.
	
	**Exclusive breastfeeding with stopping early **- the infant started out with exclusive breastfeeding (> 1 day) and then stopped breastfeeding before 12 weeks. Exclusive breastfeeding defined as infant given **only** breast milk with or without prescribed medicines. Infants were excluded from this category if > 1 day of non-exclusive breastfeeding occurred between 0 and 12 weeks.

Pregnancy complication was defined using information documented in the antenatal card. It included any of the following: anaemia, hypertension, eclampsia, sexually transmitted infection, vaginal bleed, pre-term labour, amniocentesis, TB, diarrhea, pneumonia, thrush, skin lesions, fever, excessive weight loss or gain, abnormal pap smear, fever of unknown origin, any other infection.

Postpartum complication was defined using information documented in the hospital medical record included endometritis, fever, post-partum haemorrhage, eclampsia, sepsis and mastitis.

### Data analysis

Data were entered into MS ACCESS using double data entry at a central site (MRC Durban). After validation, databases were exported to SAS version 9.1 (SAS Institute Inc., Cary NC, USA) for data management and analysis. HIV-positive and -negative women were compared using χ^2 ^tests for categorical variables (Fisher exact test if expected cell count < 5) and t-tests or Wilcoxon rank sum tests for normally and non-normally distributed continuous variables respectively. We identified whether an infant was "ever" or "never" breastfed. Table 2 explains the cross-sectional and longitudinal feeding variables generated during analysis. The proportion of HIV-positive women practicing EBF was calculated of those HIV-positive women who reported any breastfeeding, while for HIV-negative women the denominator was all negative women. To examine associations between feeding variables and infant HIV-free survival we fitted Cox proportional hazards models, using the midpoint between the last negative and first positive test as the time of infection and Efron's method for adjusting for tied survival times. We excluded HIV-positive infants at 3 weeks as early transmission is not dependent on feeding. We verified that the proportionality of hazards assumption holds. Sample size was too small in each feeding group to do analysis that explained the differences in feeding practices between HIV-positive and -negative women. To compare with the South African Demographic and Health Survey we also looked at cumulative assessment of repeated measures of feeding at birth, 3, 5, 7, 9 and 12-16 weeks to obtain proportion ever breastfed, exclusively breastfed (0-12 weeks) and not breastfed (0-16 weeks). For cross sectional analysis on the association between feeding variables and HIV-free survival feeding practices at 5 weeks were chosen, on the assumption that by 5 weeks feeding practices would have stablised.

### Ethics

Nelson R Mandela Medical School Research Ethics Committee approved the cohort study protocol (20 November 2002, Ref: E095/02). The Institutional Review Board, Columbia University granted approval for this analysis. All participating women had signed consent forms.

## Results

Six hundred and sixty-five HIV-positive and 218 HIV-negative women completed the recruitment interview and 586 (88%) HIV-positive and 197 (90%) HIV-negative women remained in the study at 3 weeks. At 36 weeks, 208 of the 883 participants enrolled in the study (21% of HIV-positive and 18% of -negative women) were lost to follow-up. HIV-positive women lost to 36-week follow-up had more advanced disease [log viral load 3.9 copies/ml (SD 0.79) vs. 3.7 copies/ml (SD 0.65), p = 0.005], were poorer [socio-economic-score -1.51 (Q1-Q3: -1.96-1.51) vs. 0.158 (Q1-Q3: 1.39-1.45), p < 0.05] and had less social support (16% disclosed their HIV status vs. 53%, p < 0.0001) compared with positive women remaining in the study. HIV-negative women lost to follow-up were similar in all respects except income, to women remaining in the study [ZAR715 (Q1-Q3: 550-1000) vs. ZAR1000 (Q1-Q3: 650-1750) respectively, p < 0.05 ].

### Characteristics of the study population

Table 3 describes the study population. Knowledge about breast milk HIV transmission differed by site amongst HIV-positive women. Reported quality of counseling was poor. Counseling score was not significantly associated with maternal knowledge about HIV transmission. Of the entire study population, 95% were ever BF from 0 to 12 weeks, no infants were exclusively breastfed from 0 to 12 weeks and 4% were never breastfed from 0 to 16 weeks.

**Table 3:** Description of study population by HIV status

	HIV-positive		HIV-negative		p value^h^
**Social-demographic factors**	**n**	**No (%)**	**n**	**No (%)**	

Mother's age in years^a^	662	25 (21-29)	217	23 (19-28)	0.003

More than 7 yrs (primary school) education	647	369 (57.0)	213	133 (62.44)	0.28

Married	665	116 (17.4)	218	42 (19.3)	0.54

Household income (ZAR/month) ^a^	553	700 (400-1200)	172	910 (640-1600)	< 0.0001

Socio-economic score^a,c^	640	-0.298 (-1.6-1.4)	213	0.273 (-1.6-1.97)	0.08

Ever disclosed HIV status	665	275 (41.4)	218	9 (4.1)	< 0.0001

Discussed infant feeding with someone other than health staff	648	186 (28.7)	216	71 (32.9)	0.25

Knew about MTCT in general	528	419 (79.4)	197	159 (80.7)	0.69

Knew about MTCT though breastfeeding	661	454 (68.7)	215	158 (73.5)	0.18

Site	665		218		

Paarl		149 (22.4)		51 (23.4)	

Rietvlei		191 (28.7)		74 (33.9)	

Umlazi		325 (48.9)		93 (42.7)	

**Health system factors**					

Counseling score^a,d, h^	663	24 (9-28)	215	0 (-4-0)	

**Medical factors**					

Log viral load ^b,e^	553	3.75 (0.7)			

No. ANC visits ^a^	655	5 (3-8)	213	5 (3-7)	

Pregnancy complications^f^	665	211 (31.7)	218	68 (31.2)	0.88

Post-partum complications^g^	665	145 (21.8)	218	33 (15.1)	0.03

Type of delivery	658		218		0.60

Vaginal		455 (69.2)		149 (68.4)	

Elective C/S		73 (11.1)		23 (10.6)	

Emergency C/S		130 (19.8)		46 (21.1)	

**Infant factors**					

Nevirapine to baby	609	596 (97.9)			

Baby's birth weight (g)	651	3016 (547)	211	3088 (527)	0.15

**Infant feeding**					

Feeding intention antenatally	653		210		< 0.0001

Exclusive formula feeding		309 (47.3)		19 (9.0)	

Exclusive breastfeeding		313 (47.9)		142 (67.6)	

Mixed breastfeeding		28 (4.3)		49 (23.3)	

**a**: median (Q1-Q3) One ZAR = approx 14US cents **b: **mean and standard deviation

**c**. Socioeconomic score (SES) - estimated using principal component factor analysis using six household assets (refrigerator, radio, television, stove, telephone/cell phone, car) and questions about food security. A weighted average was produced - items with greater variability (e.g. television) contributed to more score than items with lesser variability (e.g. radio). High socioeconomic score denotes people with more assets and food security

**d**. Counseling score - a composite measure of reported antenatal infant feeding counseling. For *HIV-positive women: *was ever discussed antenatally? (+4 if yes, -4 if no and 0 if don't know), number of times discussed (0 -none, 1 - once only, 2 - twice, 3-3 times and 4 if > 3 times) and whether the following topics were mentioned: risks of MTCT and breastfeeding (+4 if yes), different formula feeding and breastfeeding options (+4 if yes), risk of giving formula feeds (+4 if yes),, how to make best feeding choice (+4 if yes), if the mother intended to breastfeed, then avoiding mixed feeding and stopping breastfeeding early (+4 for each), how women were helped to make a choice - if women were helped to make an appropriate choice (score = +12); if health staff recommended a suitable option (score = +8); if little/no help or guidance provided with choice (score = +4). If health staff simply told women to breastfeed, score = -4. Thus maximum score was +44 and minimum was -8. For *HIV-negative women *the scores were as follows: if the counsellor reportedly discussed the risks of giving formula feeds (+4), advised against mixed feeding (+4), discussed the risks of MTCT (-4), discussed different formula feeding options (-4), advised the mother to stop breastfeeding by 6 months (-4) and discussed feeding options, helping the mother to make a choice (-4). Thus the maximum score was +8 and minimum was -16

**e: **Maternal HIV viral determined using finger-prick dried blood spots on Guthrie cards collected during the 3 and 36 week home visit. Mean maternal viral load was computed when both 3 and 36 week maternal viral load were available; otherwise maternal viral load was determined using whichever of the two was available. In cases where a mother recorded as being HIV-positive had no detectable viral load, a repeat laboratory enzyme-linked immunosorbent assay was carried out [Uniform 2 HIV-1 Assay (bioMe'rieux) followed by Biorad HIV-1 Assay (Hercules, California, USA)]

**f**. Pregnancy complication as documented in the antenatal card, including any of the following: anaemia, hypertension, eclampsia, sexually transmitted infection, vaginal bleed, pre-term labour, amniocentesis, TB, diarrhea, pneumonia, thrush, skin lesions, fever, excessive weight loss or gain, abnormal pap smear, fever of unknown origin, any other infection

**g**. Postpartum complication in hospital (endometritis, fever, post-partum haemorrhage, eclampsia, sepsis, mastitis)

**h**. p-Value only reported if comparison between HIV and positive women is sensible e.g. the counseling score is made up of different elements for HIV-positive versus negative women and thus no p-value is reported

### HIV-positive women: Feeding intention and 0-36 week practices

There were significant differences in HIV-positive women's feeding practices by site: BF initiation was commonest in Umlazi [226 (70%) vs. 72 (32%), Rietvlei and 37 (25%), Paarl], whilst FF initiation was commonest in Paarl [110 (75%) vs. 112 (58%), Rietvlei and 94 (21%), Umlazi], p < 0.001.

At 3 weeks, 309 (53%) HIV-positive women practiced any breastfeeding, of which 130 (42%) practiced EBF. By weeks 7 and 12 EBF rates dropped to 67 (30%) and 35 (18%) amongst breastfeeding HIV-positive women.

Amongst MBF HIV-positive women at week 3, 131 (73%) practiced PredBF and 48 (27%) practiced ParBF; by week 12, ParBF prevalence almost doubled with a consequent drop in PredBF [75 (48%) ParBF and 80 (52%) PredBF), due to the introduction of cereals into infants' diets (92%, 46% and 60% of MBF women in Paarl [n = 13], Rietvlei [n = 48] and Umlazi [n = 88], respectively).

Two hundred and seventy one (47%) HIV-positive women reported NBF, of which 181 (66.8%) reported feeding glucose, water, cereals, vegetables and fruit cereals in addition to commercial infant formula (MFF) from 3 weeks and increasingly thereafter (Table 3).

### Infant outcome amongst HIV-positive women

One hundred and fifty-six HIV-exposed infants were HIV-positive (n = 89) or had died (n = 67) by 36 weeks. Of the 67 who died, 50% died by 3 weeks and 75% by 13.5 weeks. Cross-sectional and longitudinal data showed poorer HIV-free survival with breastfeeding when IMR is low and poorer HIV-free survival with stopping breastfeeding early or MBF (mainly ParBF) when IMR is high (Table 4). Using longitudinal feeding data from each setting, the hazard of postnatal HIV or death (by 9 months) was highest amongst infants who avoided all breastfeeding in Rietvlei [HR 5.6 (95% CI 1.8,17)] followed by MBF infants in Paarl [HR 4.3 (95% CI 1.2,16.0) - and by infants who avoided all breastfeeding in Umlazi[4.0 (95% CI 1.2,13.7)] and MBF infants in Rietvlei [HR 2.7 (95% CI 1.0,7.2)] compared with the referent group (infants who avoided all breast milk in Paarl) - Table 2. In the two sites with higher IMR, our longitudinal data suggests that exclusive breastfeeding followed by stopping breastfeeding by 12 weeks was safer than avoiding all breastfeeding (Table 4 - in Rietvlei HR for avoiding BF was 5.6 (95% CI 1.8, 17) versus 2.8 (95% CI 0.6, 13.1) for EBF and stopping by 12 weeks. Similarly for Umlazi, HR for avoiding BF was 4.1 (95% CI 1.2, 13.7) versus 1.9 (95% CI 0.7, 5.2) for EBF with stopping by 12 weeks). The probability of HIV or death was 10.7% ±3% (mean and standard deviation) amongst infants who avoided all breastfeeding in Paarl, and 46% ±11% amongst infants in Rietvlei who stopped breastfeeding by 12 weeks (p < 0.001).

**Table 4:** Infant outcome by site and feeding practice

Feeding practices measured at 5 week visit*		HR for infant HIV or death amongst HIV-exposed infants		Longitudinal feeding practice (0-12 weeks)^#^		HR for postnatal HIV or infant death amongst HIV-exposed infants	
	**N**	**Total no. HIV/dead in each area (%)**	**HR (95% CI)**		**N**	**Total no. HIV/dead in each area (%)**	**HR (95% CI)**

**Paarl (IMR 40/100)**		**20**		**Paarl**			

EFF	48	3 (6.2%)	**Referent HR = 1**	Avoiding BF (NBF)	98	4 (4.1%)	**Referent HR = 1**

EBF	15	4 (26.7)	4.7 (1.0, 20.9)	EBF and stopping before 12 weeks	7	1 (14.3%)	1.9 (0.2, 1.5)

MBF	15	5 (33.3)	5.7 (1.4, 23.9)	MBF(l)	10	3 (30%)	4.3 (1.2, 16.0)

MFF	56	8 (14.3)	2.4 (0.6, 8.9)				

**Rietvei (IMR 99/1000)**		**46**		**Rietvei**			

EFF	14	2(14.3%)	6.6 (1.7, 25.4)	Avoiding BF (NBF)	66	14 (21.2%)	5.6 (1.8, 17)

EBF	19	7 36.8%)	2.3 (0.4, 14.0)	EBF and stopping before 12 weeks	11	3 (27.3%)	2.8 (0.6, 13.1)

MBF	64	20 (31.2%)	5.6 (1.6, 18.7)	MBF(l)	38	7 (18.4%)	2.7 (1.0, 7.2)

MFF	63	17 (27%)	4.7 (1.4, 16.1)				

**Umlazi (IMR 60/1000)**		**51**		**Umlazi**			

EFF	41	8 (19.5%)	1.2 (0.1, 11.9)	Avoiding BF (NBF)	44	7 (15.9%)	4.0 (1.2, 13.7)

EBF	14	1 (7.1%)	3.3 (0.8, 12.5)	EBF and stopping before 12 weeks	8	1 (12.5%)	1.9 (0.7, 5.2)

MBF	84	24 (28.6%)	5.0 (1.5, 16.5)	MBF(l)	29	4 (13.8%)	2.1 (0.6, 6.7)

MFF	55	18 (32.7%)	5.8 (1.7, 19.7)				

***Footnote: ***EFF = exclusive formula feeding; EBF = exclusive breastfeeding; MBF = mixed breastfeeding; MFF = mixed formula feeding (no breast) NBF = avoiding all breastfeeding. * Feeding practices defined as per Table 2 and measured over previous 96 hours at 5 week visit. Note feeding data not available on all infants who died or were lost to follow-up, thus numbers in table are slightly less than the total expected (expected N = 883 total sample, 665 HIV-exposed infants and N = 156 HIV infected or died). Note: Background IMR per site as follows; Paarl IMR 40/1000; Rietvlei IMR 99/1000 and Umlazi IMR 60/1000 at the time of the study. The numbers of women practicing longitudinal exclusive breastfeeding between 0-12 weeks was very low; thus no data on this group are presented under the longitudinal feeding data in Table 4.

### HIV-negative women: Feeding practices

**Figure 1:**
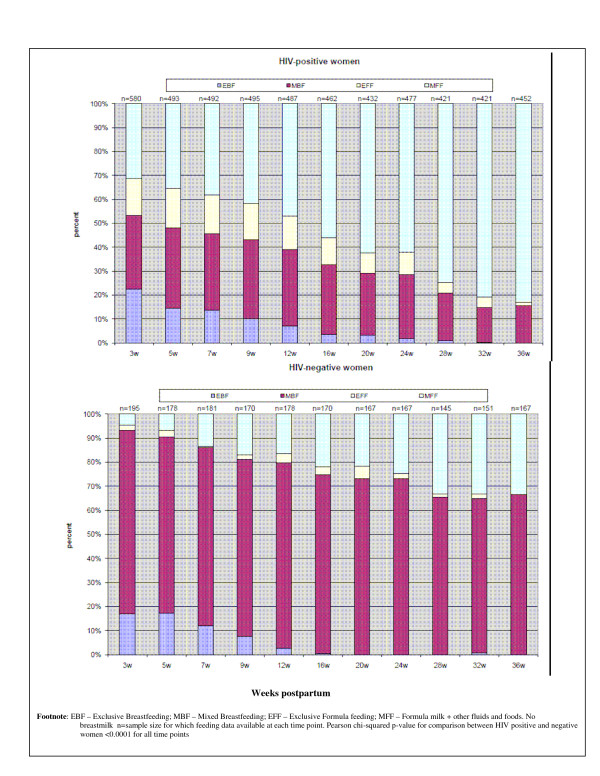
**Feeding practices amongst HIV-positive and negative women**.

Although 95% [48 (94%) Paarl, 67 (92%) Rietvlei and 89 (96%) Umlazi] of HIV-negative women initiated BF in hospital, at weeks 3, 12, 20 and 36 respectively, only 93%, 80%, 73% and 66% continued BF. At weeks 3 and 12, only 33 (17%) and 5 (3%) of HIV-negative women practiced EBF (Figure 1). None were EBF at 20 weeks. MBF was common (3-24 weeks and beyond). At week 3, 97 (65%) practiced PredBF; by week 12 the switch to partial breastfeeding had occurred in 96 (70%). By 9 months, 56 (33%) women had stopped BF.

### Comparing feeding amongst HIV-positive and -negative women

HIV-positive and -negative women had similar numbers of antenatal counseling sessions and postnatal clinic visits. Breastfeeding HIV-positive women were significantly more likely to practice EBF than HIV-negative women at 3, 5, 7, 9, 12 and 24 weeks [OR respectively = 2.4 95% CI 1.7, 3.3), 1.6 (95% CI 1.1, 2.6) 2.2 (95% CI 1.4, 3.4), 2.7 (95% CI 1.5, 4.9), 5.2, (985% CI 2.1-13) and 2.2, (95% CI 1.6-2.7)] - see Figure 1. Amongst MBF women, HIV-negative women were significantly more likely to use commercial infant formula than HIV-positive women at 3, 12, 16 and 24 weeks [OR respectively: 2.0 (95% CI 1.0, 4.0), 1.3 (1.0, 1.8), 1.4 (1.1, 1.8), 1.7 (1.1, 2.4).

## Discussion

We demonstrate sub-optimal feeding practices amongst HIV-positive and -negative mothers in PMTCT sites in the first two years after implementation of the national PMTCT programme.

### Feeding amongst HIV-positive women and child survival

In the first 6 months of life few 7-42% of breastfeeding HIV-positive women (in the three study sites) practiced EBF. By 12 weeks ParBF, which has a higher mortality risk than EBF or PredBF [1] was more common than PredBF.

Although at 3 weeks 271 HIV-positive women avoided all breastfeeding, 67% of them fed their infants formula milk and other nutritive and non-nutritive liquids and solids. This finding was unexpected and showed that although NBF seemed easier to implement than EBF, few women who avoided breastfeeding practiced EFF in the first six months. To our knowledge this practice, which we have called mixed formula feeding (MFF), has not been reported in a high HIV-prevalence resource-limited setting previously. Data from the UK shows that after adjusting for confounders EFF, compared with MFF, reduced the risk of hospital admission for diarrhea or lower respiratory tract infections in formula-fed babies, but this effect was not statistically significant. (adjusted OR = 0.59, 95% CI:0.30, 1.12 and adjusted OR = 0.85, 95% CI:0.56, 1.29, respectively) [27]. Wehypothesise that the early introduction of solids may have a stronger effect on these outcomes, especially in resource-limited settings.

Our limited analysis (Table 4) shows that MFF at 5 weeks carried a higher risk of subsequent HIV or death compared with EFF in two of the three sites. Women practicing EFF should meet specified criteria and use a suitable, nutritionally adequate breast milk substitute, which in the South African setting is commercial infant formula [9,28]. We hypothesis that in Paarl (well-resourced commercial farming area) and Umlazi (peri-urban area) mothers were able to EFF appropriately; however in Rietveli (poverty-stricken area), infants receiving formula milk only received diluted or infrequent feeds making EFF more risky than MFF.

We also show that the effect of NBF or MBF on infant HIV-free survival was site-dependent, and that avoiding breastfeeding was beneficial in Paarl, but deleterious in Rietvlei and Umlazi. Based on our previous work [29] we know that NBF was appropriate in Paarl, but inappropriate (not guided by IMR or infrastructure) in Umlazi and Rietvlei. Our data on feeding practice and hazard of HIV or death confirms that in Paarl breastfeeding is inappropriate, increasing the hazard of HIV or death. However, as background IMR increases (Umlazi and Rietvlei) our data suggest that EBF is more appropriate - as NBF reduces HIV-free survival. These differences should be considered when countries decide whether health services will principally counsel and support HIV-infected mothers to breastfeed and receive ARV interventions or avoid all breastfeeding.

### Feeding amongst HIV-negative women

HIV-negative women should all practice EBF for six months and continued BF thereafter. Our study documents feeding practices worse than those documented by cross-sectional surveys in South Africa and neighbouring countries, possibly relating to the misclassification inherent in such surveys which rely on long recall periods, or use 24-hour recall and combine data from all infants aged 0 to 6 months. Cross-sectional data from the South African Demographic and Health (DHS, 2003) survey found that 87% of infants are ever BF, 12% were EBF at 0 to 12 weeks, and 20% were NBF at 0 to 4 months [30]. Our corresponding percentages are 95% ever BF, no infants EBF (using cumulative assessment of repeated measures of feeding at birth, 3, 5, 7, 9 and 12 weeks), and 4% NBF 0 to 4 months.

Data from Botswana, Namibia and Zimbabwe show higher EBF rates under six months compared with our data (34%, 19% and 22%, respectively) [31]. Despite the high HIV prevalence in South Africa [32], HIV-negative women constitute at least 70% of the population; thus, the poor feeding practices amongst HIV-negative women are of grave concern for overall child health.

### Implications for routine infant feeding counseling services in South Africa and similar countries

Our data show that HIV-positive women (who were exposed to the PMTCT programme as vertically implemented and who received specific infant feeding counseling antenatally and follow-up postnatally [13]) reported less use of commercial infant formula by MBF mothers and more exclusive feeding compared with HIV-negative women, (who were followed up by child health care services). These findings are similar to cross-sectional findings of Orne-Gliemann et.al., Zimbabwe [33], and Magezi, Uganda [21], who document earlier commencement of MBF and poorer adherence to feeding recommendations, respectively amongst HIV-negative compared with positive women. Furthermore, we show that in 2001-2003 the EBF prevalence at 12 weeks was low - 7.2% and 2.8% amongst breastfeeding HIV-positive women and negative women respectively. These data are corroborated by the recent Lancet publication on breastfeeding promotion which showed a very low baseline EBF prevalence in South Africa with subsequent increase following an EBF home-based intervention [23] [EBF prevalence at 12 weeks in control and intervention clusters respectively were 19 (4%) versus 41 (8%) in South Africa (Prevalence ratio intervention:control:1.98, 95% CI 1.30, 3.02) compared with 94 (23%) versus 300 (77%) in Burkina Faso (Prevalence ratio I:C: 3.27, 95% CI, 2.13, 5.03); 125 (34%) versus 305 (77%) in Uganda (Prevalence ratio I:C: 2.30, 95% CI 2.00, 2.65)]. We hypothesise that South African HIV-negative women receive ambiguous feeding messages possibly as a result of four factors: Firstly PMTCT was implemented as a vertical programme in 2002; thus routine child health care providers may not have received standardized training on feeding in the context of HIV. Secondly, the routine child health service promotes commercial infant formula as part of the Protein Energy Malnutrition scheme. Thirdly, the Code of Marketing of BreastmilkSubstitutes[23] was not legislated in South Africa in 2002. Fourthly, infant feeding counseling is the weakest link in the routine child health programme [34]. HIV-positive women, on the other hand, received infant feeding counseling by trained PMTCT counselors and this may have clarified ambiguous messages. Thus, the South African poor infant feeding practice problem seems to be systemic. A recent breastfeeding summit in South Africa (22-23 August 2011) adopted the Tshwane Declaration of Support for Breastfeeding, which recommends the removal of free commercial infant formula as part of the PMTCT programme and the promotion of exclusive breastfeeding to optimise child survival [35]. The effects of this renewed commitment to breastfeeding at a national level will need to be monitored.

### Strengths and limitations of our analysis

Our study and analysis had several limitations: Maternal HIV status was determined by the routine PMTCT programme; however confirmatory ELISA tests on women with undetectable viral loads and on a sub-sample of negative women were run. Our cross-sectional and longitudinal feeding variables allowed fewer lapses than that allowed by Coovadia et.al [5], as we had fewer data collection points and did not use diaries, thus we assumed that even one non-breast milk day may be a marker of many more non-breast milk days. Our variables are, however, more stringent than those used by Coutsoudis et.al [6] and Illiff et.al [7] who, in the first 12 weeks of life assessed feeding cross-sectionally at 1, 6, and 12 weeks and at birth, 6 and 12 weeks, respectively. However, the fundamental idea of our definitions are similar to those used by Coovadia et.al. [5], and the main methodological differences between our study and previous publications are due to differing data collection points. Analysis of our infant feeding data was complex due to repeated measures, unequal observation times, reliance on 4-day recall to categorise feeding patterns during each visit, and loss to follow-up. Given these complexities we present a simplistic analysis which could lead to an inflation of the type I error rate; more complex analyses however, would have resulted in loss of much of the sample. Furthermore, the consistency of the point estimates in Table 4 is reassuring.

## Conclusions

Infant feeding messages need to be clear, and these should be integrated within all routine child health services where HIV-positive women need to be seen as a group with special needs, as suggested by the Global Strategy for IYCF [36]. Infant feeding interventions should include HIV-negative mothers who constitute the majority of the world's mothers, and whom we will fail if our efforts excessively prioritise HIV-positive women. The effect of the Tshwane Declaration (South Africa, August 2011) that renews support for breastfeeding needs to be monitored to ensure that the low rates of breastfeeding (and exclusive breastfeeding in particular) measured in 2002-3 and 2006-8 will be reversed with a corresponding gain in child survival.

## Abbreviations

EBF: Exclusive Breastfeeding; HIV: Human Immunodeficiency Virus; IMR: Infant Mortality Rate; MBF: Mixed Breastfeeding; MRC: Medical Research Council; NBF: Not Breastfeeding; ParBF: Partial Breastfeeding; PMTCT: Programme to Prevent Mother to Child Transmission of HIV; PredBF: Predominant Breastfeeding; RR: Risk Ratio; WHO: World Health Organization

## Competing interests

The authors declare that they have no competing interests.

## Authors' contributions

AEG participated in the conceptualisation of the paper, analysis of the data and writing of the paper. TD, DJ, M Colvin and M Chopra participated in the conceptualisation of the study, implementation of the study, and writing of the paper. DS participated in the writing of the paper. LK participated in the conceptualisation of the paper, provided guidance on the analysis of the data and participated in the writing of the paper. All authors read and approved the final manuscript.
